# Integrating the Sensation–Emotion–Cognition (SEC) Model into Tinnitus Care: A Preliminary Exploratory Study of a Comprehensive Tinnitus Management Protocol

**DOI:** 10.3390/audiolres16020043

**Published:** 2026-03-09

**Authors:** María del Carmen Moleón González, Farzon Danesh, Ali A. Danesh

**Affiliations:** 1 Instituto de Investigación Biosanitaria ibs.GRANADA, Hospital Universitario Clínico San Cecilio, 18012 Granada, Spain; mcmoleon@ugr.es; 2 Labyrinth Audiology, Boca Raton, FL 33486, USA; fdanesh2015@fau.edu; 3 Department of Communication Sciences and Disorders, Florida Atlantic University, Boca Raton, FL 33431, USA

**Keywords:** tinnitus, SEC model, cognitive behavioral therapy, audiological management, distress

## Abstract

Background: Tinnitus, the perception of sound in the absence of an external source, is a prevalent condition that can substantially affect physical and mental health. Although tinnitus is not typically curable, it is often manageable with structured, multidisciplinary care. This pilot research describes the Sensation–Emotion–Cognition (SEC) model, a practical audiological framework developed by Danesh et al. that targets three core dimensions of the tinnitus experience. Methods: We integrate findings from an exploratory retrospective cohort and a prospective expansion study. The SEC protocol included sound therapy, counseling and relaxation training, and cognitive behavioral therapy (CBT) delivered through either unguided, module-based internet CBT, clinician-guided module-based internet CBT, or six therapist-led CBT sessions. The objective was to evaluate whether this multifactorial approach is associated with reductions in tinnitus-related distress. Results: In this prospective study, preliminary results from 16 participants who completed the study were associated with significant pre–post changes in tinnitus-related outcomes: 4C management confidence increased from M = 30.38 to 60.19 (*p* < 0.001; Cohen’s dz = 1.04), and SAD-T emotional distress decreased from M = 4.75 to 2.38 (*p* = 0.001; Cohen’s dz = 0.99). Conclusions: These findings suggest the potential value of an integrated management strategy; however, given the single-group pre–post design and attrition, the results should be interpreted as exploratory and warrant confirmation in larger controlled trials.

## 1. Introduction

Tinnitus is defined as the perception of sound in the absence of external acoustic stimulation [1,2]. It is described in a wide variety of ways in terms of pitch, loudness, and sound quality, and psychoacoustic studies have highlighted large inter-individual variability in the tinnitus percept [3,4]. Large population-based data indicate that tinnitus affects around 14% of adults worldwide and is perceived as a major problem by approximately 2–3% [5,6]. Although most individuals with tinnitus also present with some degree of hearing loss, hearing loss is not sufficient to cause tinnitus, and the association between audiometric thresholds and subjective tinnitus loudness is typically modest [7,8]. For example, a large UK clinic study reported that tinnitus loudness increased by only 0.022 units for each 1 dB increase in pure-tone average [7]. Despite significant advances in neuroscience, the pathophysiology of tinnitus remains only partially understood. At present, there is no treatment that reliably eliminates the tinnitus percept [1,2,9].

Modern conceptualizations recognize that tinnitus-related distress is not solely an audiological phenomenon (Sensation) but is strongly influenced by emotional reactions (Emotion) and maladaptive cognitions and thoughts (Cognition). Multiple reviews and meta-analyses have documented robust associations between tinnitus and anxiety, depression, insomnia, and stress-related conditions [10]. Contemporary guidelines, therefore, recommend multimodal, psychologically informed management, with cognitive behavioral therapy (CBT) and internet-based CBT (iCBT) representing core evidence-based interventions for bothersome tinnitus [3,11,12,13,14,15,16,17]. In association with this background, the Sensation–Emotion–Cognition (SEC) model [18] was developed as a patient-friendly and clinician-guided framework that explicitly targets these three interlinked dimensions of tinnitus distress.

## 2. The Sensation-Emotion-Cognition (SEC) Model

The SEC model simplifies the complex experience of tinnitus into three interlinked components, each requiring a specific therapeutic intervention.

### 2.1. Sensation (Sound Therapy)

The first component addresses the tinnitus sound itself. The goal is to prevent the auditory system and attention networks from becoming hyper-focused on the perception, especially in quiet environments. This is achieved through sound therapy, which enriches the acoustic environment. Patients use sound generators or hearing aids that provide gentle, low-level background noise. This competing auditory input reduces the relative intensity and salience of the tinnitus, facilitating habituation and allowing the sound to recede into the background of awareness over time. The current report does not include sensory stimulation by modalities such as acoustic-electrical neuromodulation. However, the same principles apply if such modalities are utilized [3].

### 2.2. Emotion (Counseling and Relaxation)

The second focus targets the emotional distress, anxiety, and autonomic nervous system reactions often provoked by the tinnitus signal. The aim is to reduce the emotional and physiological response. Through professional counseling, psychoeducation, and specific relaxation strategies, patients learn to reinterpret the tinnitus signal as non-threatening. By weakening the association between the sound and a perceived danger, the stress response diminishes, leading to improved daily functioning [14,19].

### 2.3. Cognition (Cognitive Behavioral Therapy—CBT)

The final component addresses the negative, maladaptive thought patterns that accompany chronic tinnitus such as catastrophic thinking or hopelessness. CBT is utilized to identify, question, and replace these negative thoughts with more balanced and realistic alternatives. Techniques include examining evidence for and against tinnitus-related thoughts, running behavioral experiments, and developing helpful alternative statements [3,20].

The SEC model aligns with established neurophysiological and psychological frameworks of tinnitus distress, including aberrant auditory gain and salience [21], maladaptive emotional reactivity, and dysfunctional cognitive appraisal. Drawing on fear-avoidance, cognitive-behavioral, and predictive coding models, SEC provides a pragmatic clinical framework that translates these mechanisms into coordinated therapeutic targets.

## 3. Methods

The pilot evidence presented here reports findings from two studies: (1) an exploratory, retrospective study assessing the foundational SEC audiological protocol (Sensation and Emotion components) combined with unguided computer-based iCBT, and (2) a prospective two-stage expansion study with structured intervention (including Sensation, Emotion, and Cognition components) utilizing clinician-guided iCBT and six-session online face-to-face CBT. This prospective study used a single-group pre–post design. Baseline characteristics between completers and non-completers were examined descriptively.

Primary outcomes were selected as a priori to map directly onto the treatment targets of the SEC model and guided CBT: tinnitus-related distress (SAD-T) and tinnitus coping self-efficacy (4C). Both instruments have published psychometric support and were chosen to be sensitive to change over the intervention.

This retrospective study was conducted in accordance with the Declaration of Helsinki and complied with all applicable regulations, including HIPAA requirements. The data were collected in a private practice setting where the participants/patients had agreed that their data would be used for research purposes anonymously. The authorization from participants was obtained on two occasions. First, when the evaluation started at the private practice, and second, from the CBT provider. De-identified and anonymous data from the participants were used after their authorization.

### 3.1. Participants

**Figure 1:**
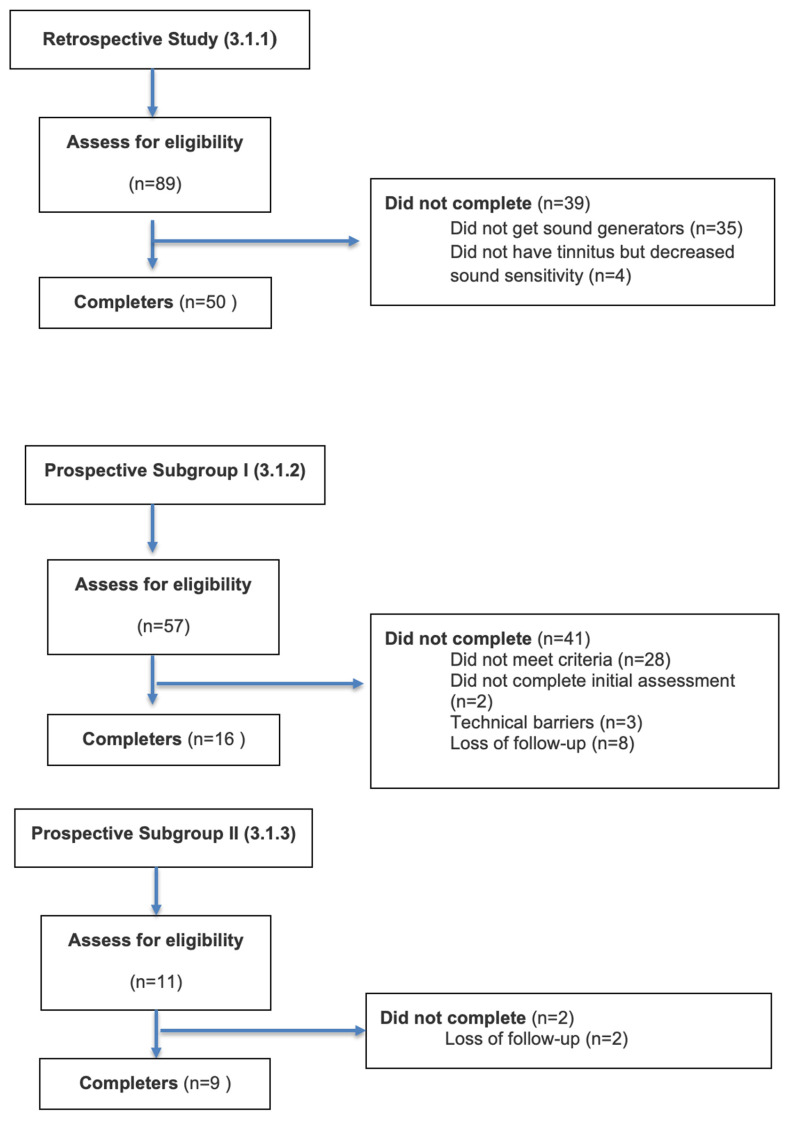
Flow of participants through the retrospective and prospective studies.

Three categories of participants are described here (Figure 1):

- This retrospective study initially recruited 89 participants from a tinnitus clinic, and from these, 50 completed the SEC model management plan. The participants received monaural or binaural sound generators (sensation), counseling and relaxation techniques (emotion), and an unguided computer-based CBT program (cognition). The mean age (M_age_) of the 50 participants included was 57.8 years (range: 20–87 years).
- Prospective subgroup I initially included 57 participants from the same tinnitus clinic, who enrolled in the study; however, data from 16 of the 57 original participants met the inclusion criteria (i.e., they had the three components of the SEC model: sensation, emotion, and cognition). Reasons for non-completion included incomplete participation in CBT modules, loss to follow-up, and technical barriers when accessing the online platform. The only difference with the first group was that the participants had instructions and guidance provided by a qualified tinnitus CBT therapist online. The therapist instructed the participants on how to navigate the iCBT program and answer their questions (i.e., guided iCBT). The mean age (*M_age_*) of the 16 participants included in this preliminary analysis was 56.56 years (range: 24–73 years). Given the high attrition rate, baseline characteristics of completers and non-completers were compared descriptively, where data were available to assess potential selection bias.
- Prospective subgroup II included 11 participants from the same clinic, who received the three components of the SEC model, except that their CBT delivery was six sessions of face-to-face meetings online with a qualified tinnitus CBT specialist. The mean age (*M_age_*) of the 11 participants included was 56.88 years (range: 45–75 years).

All participants were diagnosed with chronic subjective tinnitus, defined as at least 6 months’ duration. The specific inclusion criteria for participants in the SEC protocol were the use of sound generators or hearing aids, completion of one of the CBT delivery models depicted above, and participation in counseling and relaxation training. A minimum symptom severity threshold was applied, defined as a Tinnitus Handicap Inventory (THI) score ≥ 38. Exclusion criteria included severe psychiatric disorders, cognitive impairment, and ongoing alternative or experimental tinnitus treatments during the study period.

Missing data occurred due to participant dropout, incomplete questionnaire responses, or technical issues. Given the exploratory nature of the study and the limited sample size, analyses were conducted using a complete-case approach, including only participants with available data for the relevant outcomes. No data imputation procedures were applied.

### 3.2. Intervention: The Full SEC Protocol

All participants received the core Sensation and Emotion components of the SEC model, including sound generators/hearing aids and counseling. The Cognition component (CBT) was delivered using one of three methods used in clinical settings, as described below:

1. Computer-based CBT (unguided iCBT): Participants completed standardized CBT modules independently, without direct clinician support.
2. Computer-guided CBT (guided iCBT): Participants completed the same CBT modules but received structured clinician guidance, including scheduled one-on-one sessions to support engagement, clarify content, and reinforce cognitive and behavioral strategies.
3. Face-to-face CBT: Participants received six CBT sessions through in-person online visits with a trained clinician, following CBT principles consistent with tinnitus-focused cognitive restructuring and skills training.

The total duration of the SEC intervention varied across participants depending on the CBT delivery model, with interventions typically spanning several weeks to months. Participants were instructed to use these devices on a daily basis; however, objective measures of average daily usage hours or weekly frequency were not systematically recorded. Counseling was delivered as part of routine tinnitus management and included education on tinnitus mechanisms, coping strategies, and relaxation techniques. The number and duration of counseling sessions varied according to individual clinical needs and were not standardized.

In the unguided and guided iCBT groups, standardized online CBT modules were completed; however, detailed adherence metrics such as module-specific completion times and login frequency were not consistently available. In the face-to-face CBT group, participants received six CBT sessions delivered by a qualified clinician. Participant engagement was assessed primarily through completion of the intervention components. Detailed engagement metrics, including exact dropout timing and platform usage frequency, were not systematically collected.

### 3.3. Outcome Measures

Participants underwent audiological evaluation and completed tinnitus-related assessments, including the Tinnitus Retraining Questionnaire (TRQ), Screening for Anxiety and Depression in Tinnitus (SAD-T), and the Four Components of Tinnitus Management Questionnaire (4C). Tinnitus-related distress and coping were assessed at baseline (pre-treatment) and after completion of the intervention (post-treatment). The retrospective outcome report used TRQ, and the prospective two-stage study outcomes employed SAD-T and 4C. Only participants with complete pre- and post-treatment data for the relevant outcomes are included in this report. Different validated instruments were used across cohorts because outcome collection reflected routine clinical practice and an evolution of the program evaluation over time. For interpretability, the prospective study prioritized measures mapping directly onto SEC targets (emotional distress: SAD-T; self-efficacy/management confidence: 4C).

### 3.4. Statistical Analysis

Analyses were conducted in the prospective cohort using a complete-case approach (participants with complete pre- and post-treatment data for the relevant outcomes). Pre–post changes in validated outcome measures (SAD-T and 4C) were evaluated using paired-samples t-tests. Change scores were inspected for gross deviations from normality. Two-tailed tests were applied, with statistical significance set at *p* < 0.05. Mean changes are reported with 95% confidence intervals. Effect sizes were calculated as Cohen’s dz for within-subject pre–post comparisons. As an exploratory secondary analysis, associations between changes in SAD-T (Δ post–pre) and changes in 4C (Δ post–pre) were examined using Pearson and Spearman correlation coefficients. No power calculation was performed, as the study was designed as an exploratory pilot investigation.

## 4. Results

### 4.1. Exploratory Retrospective Findings (SEC + Unguided iCBT)

The initial retrospective report (N = 50) evaluated the foundational SEC audiological management combined with unguided iCBT. Mean pre-treatment TRQ scores were 57.1, decreasing to 31.5 post-treatment. Although the TRQ does not have a universally accepted minimal clinically important difference (MCID), previous clinical studies and intervention protocols have defined treatment success using relative change criteria, most commonly a ≥40% reduction in TRQ total score. In our sample, the observed absolute reduction of 25.6 points and a relative reduction of 44.8% exceed this threshold and therefore indicate a clinically relevant improvement in tinnitus-related distress (Table 1).

### 4.2. Baseline Comparison Between Completers and Non-Completers

The baseline characteristics of participants who completed the intervention were compared with those of participants who did not complete the protocol in order to assess potential attrition-related selection bias. As shown in Table 2, completers and non-completers were broadly comparable across clinical variables. No statistically significant differences were observed in baseline tinnitus self-management confidence (4C) or tinnitus-related emotional distress (SAD-T) when analyzed (*p* = 0.156 and *p* = 0.177, respectively). These findings suggest that attrition did not appear to systematically bias the sample, although the relatively small sample size limits statistical power.

### 4.3. Prospective Outcomes Following the SEC Protocol with Guided iCBT

Preliminary analysis of 16 completers indicated significant improvement across the assessed outcome measures. SAD-T scores decreased from pre- to post-treatment (M = 4.75 to 2.38; t(15) = −3.97, *p* = 0.001; Cohen’s dz = 0.99). Conversely, 4C management confidence scores increased significantly (M = 30.38 to 60.19; t(15) = 4.17, *p* = 0.0008; Cohen’s dz = 1.04). These findings suggest that the full SEC protocol, including guided iCBT, may represent a structured approach associated with reductions in tinnitus-related emotional distress and improvements in tinnitus management confidence in this preliminary cohort (Table 3).

Changes in emotional distress were not significantly correlated with changes in management confidence (Pearson r = −0.216, *p* = 0.422; Spearman ρ = −0.106, *p* = 0.696) (Table 4), suggesting that improvements across emotional and cognitive domains may occur relatively independently in this preliminary cohort.

### 4.4. Prospective Outcomes Following the SEC Protocol with Face-to-Face CBT

Preliminary analysis of participants receiving the SEC model with face-to-face CBT indicated clinically meaningful improvements across the assessed outcome measures, although sample size was limited by data completeness. For emotional distress, SAD-T scores decreased from pre- to post-treatment (M = 4.11 to 1.67), corresponding to a mean reduction of −2.44 points (SDΔ = 2.01). This pattern indicates a notable reduction in tinnitus-related emotional distress over the intervention period. In addition, 4C management confidence increased from pre- to post-treatment (M = 24.43 to 42.86), corresponding to a mean improvement of +18.43 points (SDΔ = 18.65) (Table 5).

### 4.5. Exploratory Comparison of Guided iCBT Versus Face-to-Face CBT on Emotional Distress (SAD-T)

To directly compare changes in tinnitus-related emotional distress across CBT delivery formats, a linear mixed-effects model with a random intercept for participants was fitted to SAD-T scores. Fixed effects included Time (pre vs. post), Group (guided iCBT vs. face-to-face CBT), and their interaction.

There was a significant main effect of Time, indicating an overall reduction in SAD-T from pre- to post-treatment (F(1,25) = 28.35, *p* = 1.61 × 10^−5^). The main effect of Group was not significant (F(1,25) = 0.41, *p* = 0.527). Importantly, the Time × Group interaction was not significant (F(1,25) = 0.006, *p* = 0.939), indicating that the magnitude of pre–post improvement in emotional distress did not differ between guided iCBT and face-to-face CBT. Estimated marginal means showed significant reductions in SAD-T in both groups (face-to-face CBT: 4.11 to 1.67; guided iCBT: 4.75 to 2.38; Table 6).

Estimated marginal means derived from the linear mixed-effects model indicated significant reductions in SAD-T scores from pre- to post-treatment in both groups. Participants receiving face-to-face CBT showed a decrease from 4.11 to 1.67 (mean change = −2.44, *p* = 0.003), while those receiving guided iCBT showed a decrease from 4.75 to 2.38 (mean change = −2.38, *p* < 0.001). The magnitude of improvement was comparable across groups (Table 7).

### 4.6. Exploratory Comparison of Guided iCBT Versus Face-to-Face CBT on Tinnitus Management Confidence (4C)

A similar linear mixed-effects model was fitted to 4C scores to evaluate changes in tinnitus management confidence. A significant main effect of Time was observed (F(1,23.59) = 16.78, *p* = 0.000425), indicating an overall increase in confidence from pre- to post-treatment. The main effect of Group was not significant (F(1,24.13) = 0.81, *p* = 0.376), and the Time × Group interaction was also not significant (F(1,23.59) = 1.47, *p* = 0.238), suggesting no statistically significant difference in confidence gains between guided iCBT and face-to-face CBT (Table 8).

Estimated marginal means showed increases in both groups. Within-group improvement reached statistical significance for guided iCBT (+29.8 points, *p* = 0.0001), whereas the increase observed in the face-to-face CBT group (+16.2 points) did not reach significance (*p* = 0.1095), noting that 4C data were partially missing in the face-to-face subgroup (Table 9).

## 5. Discussion

The preliminary findings from the full SEC protocol align with the broader evidence base for psychologically informed tinnitus management. Meta-analytic evidence consistently supports CBT as the most robust intervention for reducing tinnitus-related distress, typically demonstrating small-to-moderate effects relative to control conditions [3,15].

In the present prospective cohort (guided iCBT; N = 16 completers), emotional distress measured by SAD-T showed a significant pre–post reduction with a large within-subject effect size (Cohen’s dz = 0.99). Although cross-study comparisons should be interpreted cautiously, the magnitude of improvement is broadly consistent with contemporary internet-based CBT trials. For example, audiologist-guided iCBT has been shown to yield clinically meaningful improvements in tinnitus-related outcomes relative to monitoring conditions [22]. These results support the plausibility that integrating SEC-based counseling and relaxation strategies with guided iCBT may reduce distress by targeting psychological processes relevant to the Emotion domain [23].

Management confidence (4C) showed a substantial pre–post increase in the guided iCBT cohort, also with a large within-subject effect (Cohen’s dz = 1.04). The 4C questionnaire indexes perceived control and self-efficacy in tinnitus management, behavioral experiments, and skills consolidation [23]. While within-subject effect sizes are not directly comparable to between-group estimates from randomized trials, the observed increase is consistent with evidence that guided digital interventions can meaningfully improve self-management outcomes [16].

These prospective findings extend earlier retrospective observations, where TRQ scores decreased from 57.1 to 31.5 (absolute reduction 25.6 points; ~44.8% relative reduction) following SEC-based audiological management combined with unguided iCBT. Although the current work was not designed to directly compare guided versus unguided cohorts, the pattern of improvement is consistent with theoretical and empirical work indicating that clinician support may enhance engagement, adherence, and skill acquisition in digital CBT programs [24,25].

Importantly, in an exploratory comparison of CBT delivery formats within the SEC framework (guided iCBT versus face-to-face CBT), linear mixed-effects models indicated significant improvements over time in both emotional distress (SAD-T) and management confidence (4C), with no evidence of differential efficacy between formats (non-significant Time × Group interactions). Although estimated marginal means suggested a numerically larger increase in 4C in the guided iCBT subgroup, confidence gains did not differ statistically between modalities, and 4C data were partially missing in the face-to-face subgroup, limiting power and precision.

Changes in emotional distress were not significantly correlated with changes in management confidence, suggesting that improvements across emotional and cognitive domains may occur relatively independent of the cohort. The absence of a significant association between changes in emotional distress and changes in tinnitus management confidence suggests that these outcomes may reflect partially independent therapeutic processes. Reductions in emotional distress may occur relatively early through reassurance, normalization, and reduced threat appraisal, whereas gains in self-efficacy and management confidence may require longer-term consolidation of coping strategies and behavioral practice. This dissociation is consistent with cognitive-behavioral models in which affective relief and self-management confidence do not necessarily evolve in parallel, particularly in short-term or exploratory interventions.

A major limitation of this prospective cohort is the high attrition rate observed in the guided iCBT subgroup, in which only 16 of 57 enrolled participants completed all intervention components. Although baseline comparisons suggested that completers and non-completers were broadly comparable across most demographic and clinical variables, attrition-related selection bias cannot be excluded. Participants who completed the intervention may have been more motivated, more technologically engaged, or more able to adhere to structured behavioral programs, potentially inflating observed treatment effects. Consequently, findings should be interpreted as exploratory and may not be fully generalizable to the broader tinnitus population.

Another limitation of this study is that the integrated and multimodal nature of the SEC protocol does not allow the disentangling of the specific contributions of individual components to the observed improvements. Consequently, the observed reduction in tinnitus-related distress and increases in management confidence should be interpreted as associated with the combined SEC approach.

A key strength of the SEC model is its integrative structure, explicitly targeting sensory, emotional, and cognitive dimensions of tinnitus distress, an approach consistent with clinical guidance and the recent literature advocating multi-component interventions [3,17]. However, several limitations warrant emphasis. Sample sizes were small, particularly in the face-to-face CBT subgroup and for complete 4C data, and the prospective analyses were based on pre–post designs without randomized controls, precluding causal inference. Therefore, observed changes should be interpreted as associations rather than causal treatment effects. Future studies should include randomized or controlled designs with strategies to improve retention.

## 6. Conclusions

The Sensation, Emotion, and Cognition (SEC) model offers a comprehensive, patient-centered framework for the management of chronic tinnitus. By explicitly targeting the auditory, affective, and cognitive dimensions of the condition, the model goes beyond simple sound masking and aims to promote changes in neural processing associated with tinnitus-related distress, with the aim of reducing symptom burden and improving quality of life.

In the prospective data, participants receiving the SEC model combined with guided iCBT showed significant pre–post improvements in emotional distress (SAD-T) and management confidence (4C). When directly comparing guided iCBT and face-to-face CBT within the SEC framework using linear mixed-effects models, both formats indicated significant improvements over time, and there was no evidence of differential efficacy between delivery formats (non-significant Time x Group interactions for both SAD-T and 4C). Given the single-arm pre–post design and high attrition in the prospective cohort, these findings should be considered exploratory. Confirmation in larger studies employing controlled or randomized designs is required before causal conclusions can be drawn.

## Figures and Tables

**Table 1:** Descriptive TRQ outcomes in the retrospective SEC cohort.

Outcome Measure	Pre-Treatment Mean (Range)	Post-Treatment Mean (Range)	Absolute Change (Points)	Relative Change (%)	Clinical Significance
TRQ Total Score	57.1 (8–104)	31.5 (0–74)	−25.6	−44.8%	Clinically relevant improvement (≥40% reduction)

**Table 2:** Baseline characteristics of completers and non-completers in the prospective guided iCBT cohort.

Variable	Completers	Non-Completers	*p*-Value
Female (%)	46.9%	24%	0.17
4C pre-treatment (mean, SD)	30.38 (23.54)	21.52 (22.67)	0.156
SAD-T pre-treatment (mean, SD)	4.75 (3.28)	6.33 (4.97)	0.177

**Table 3:** Pre- and post-treatment scores on the SAD-T and 4C questionnaires for participants receiving the full SEC protocol. *Note.* Effect sizes are reported as Cohen’s dz for within-subject pre–post comparisons. “g” denotes Hedges’ g (small-sample corrected effect size).

Outcome Measure	N	Pre-Treatment Mean (SD)	Post-Treatment Mean (SD)	Mean Change (SDΔ)	95% CI (Δ)	t(df), *p*	Wilcoxon *p*	Effect Size Cohen dz (g)
SAD-T (0–12)	16	4.75 (3.28)	2.38 (3.26)	−2.38 (2.39)	[−3.65, −1.10]	t(15) = −3.97, *p* = 0.001	0.0049	dz = 0.99; g = 0.94
4C (0–100)	16	30.38 (23.54)	60.19 (27.97)	29.81 (28.58)	[14.58, 45.04]	t(15) = 4.17, *p* = 0.0008	0.0004	dz = 1.04; g = 0.99

**Table 4:** Association between changes in emotional distress and management confidence.

Variables (Δ Post-Pre)	N	Pearson r (95% CI)	*p*-Value	Spearman ρ	*p*-Value
ΔSAD-T vs. Δ4C	16	−0.216 [−0.643, 0.314]	0.422	−0.106	0.696

**Table 5:** Pre- and post-treatment scores on the SAD-T and 4C questionnaires for participants receiving the full SEC protocol and face-to-face CBT. *Note.* Effect sizes are reported as Cohen’s dz for within-subject pre–post comparisons. “g” denotes Hedges’ g (small-sample corrected effect size).

Outcome Measure	N	Pre-Treatment Mean (SD)	Post-Treatment Mean (SD)	Mean Change (SDΔ)	95% CI (Δ)	t(df), *p*	Wilcoxon *p*	Effect Size Cohen dz (g)
SAD-T (0–12)	9	4.11 (1.69)	1.67 (2.00)	−2.44 (2.01)	[−3.99, −0.90]	t(8) = −3.65, *p* = 0.0065	0.0260	dz = 1.22; g = 1.10
4C (0–100)	9	24.43 (22.66)	42.86 (27.26)	+18.43 (18.65)	[1.18, 35.68]	t(8) = 2.61, *p* = 0.0399	0.0156	dz = 0.99; g = 0.86

**Table 6:** Linear mixed-effects model results for SAD-T scores comparing guided iCBT and face-to-face CBT.

Effect	Estimate (β)	Standard Error (SE)	df	t	*p*-Value
Intercept (Face-to-face, Pre)	4.11	0.91	34.0	4.50	<0.001
Time (Post vs. Pre)	−2.44	0.72	25.0	−3.38	0.002
Group (Guided iCBT vs. Face-to-face)	0.64	1.14	34.0	0.56	0.580
Time × Group	0.07	0.91	25.0	0.08	0.939
Random effect	Variance		SD		
Participant (intercept)	5.15		2.27		
Residual	2.36		1.54		

**Table 7:** Pre- and post-treatment estimated SAD-T means by the CBT group derived from the linear mixed-effects model.

Group	Pre-Treatment Mean (SE)	Post-Treatment Mean (SE)	Mean Change (Post–Pre)	*p*-Value
Face-to-face CBT	4.11 (0.95)	1.67 (0.95)	−2.44	0.003
Guided iCBT	4.75 (0.71)	2.38 (0.71)	−2.38	<0.001

**Table 8:** Linear mixed-effects model for 4C comparing guided iCBT and face-to-face CBT.

Effect	Estimate (β)	SE	df	t	*p*-Value
Intercept (Face-to-face, Pre)	28.75	8.74	38.04	3.29	0.002
Time (Post vs. Pre)	16.21	9.32	23.96	1.74	0.095
Group (Guided iCBT vs. Face-to-face)	1.63	10.71	38.04	0.15	0.880
Time × Group	13.61	11.23	23.59	1.21	0.238

**Table 9:** Pre- and post-treatment estimated 4C means by the CBT group derived from the linear mixed-effects model. *Note.* Analyses were based on available 4C observations (3 missing values, all within the face-to-face CBT group). EMMs are model-derived estimates accounting for repeated measures and between-participant variability.

Group	Pre-Treatment Estimated Marginal Mean (SE)	Post-Treatment Estimated Marginal Mean (SE)	Mean Change (Post–Pre)	*p*-Value
Face-to-face CBT	28.8 (9.14)	45.0 (9.66)	16.2	0.1095
Guided iCBT	30.4 (6.46)	60.2 (6.46)	29.8	0.0001

## Data Availability

The data presented in this study are available on request from the corresponding author.
